# CD52 knockdown inhibits aerobic glycolysis and malignant behavior of NSCLC cells through AKT signaling pathway

**DOI:** 10.7150/jca.86511

**Published:** 2024-04-29

**Authors:** Yini Cai, Jiali Zhao, Chen Luo, Ming Fang, Yanling Yi, Yu Chen, Peng Huang, Lingmin Liao, Long Huang

**Affiliations:** 1 Department of Oncology, The Second Affiliated Hospital of Nanchang University; Jiangxi Key Laboratory of Clinical and Translational Cancer Research, 1 Minde Road, Nanchang, Jiangxi, China.; 2 Department of Ultrasound, The Second Affiliated Hospital, JiangXi Medical College, Nanchang University, Nanchang, China.; 3 Department of Yangxin People's Hospital of Hubei Province, 81 Ruxue Road, Xingguo Town, Yangxin County, Huangshi, Hubei, China.

**Keywords:** NSCLC, CD52, Proliferation, Migration, aerobic glycolysis, AKT

## Abstract

CD52 is an important functional regulator involved in the development of human cancer. In this study, the clinical significance and biological function of CD52 in the malignant behavior of non-small cell lung cancer (NSCLC) were explored. In this study, immunohistochemical (IHC) staining was performed to determine the expression pattern of CD52 in NSCLC. Loss of function assays were used to evaluate the biological functions of CD52 in NSCLC cells *in vitro* and *in vivo*. Our data indicated that the expression of CD52 was significantly elevated in NSCLC and correlated with the patient prognosis. Functionally, downregulation of CD52 expression significantly suppressed the proliferation, migration, aerobic glycolysis and tumorigenesis of NSCLC cells. Moreover, CD52 regulated aerobic glycolysis of NSCLC cells through the AKT pathway. Furthermore, aerobic glycolysis induced by 2-DG inhibited the proliferation of NSCLC cells. In conclusion, CD52 knockdown inhibited aerobic glycolysis and malignant behavior of NSCLC cells through AKT signaling pathway, which may be employed in an alternative therapeutic target for NSCLC.

## Introduction

Lung cancer is one of the malignant tumors with high morbidity and mortality, which is a threat to human health and life worldwide 1. Approximately 85% of lung cancer patients are non-small cell lung cancer (NSCLC), of which lung adenocarcinoma (LUAD) and lung squamous cell carcinoma (LUSC) are the most common subtypes 2. The lack of typical symptoms in the early stages of the disease results in approximately 75% of patients being diagnosed at advanced stage, with a low five-year survival rate 3. Chemotherapy, radiotherapy and surgery are still the main management for NSCLC 4. In the past two decades, the targeted therapy and immunotherapy of NSCLC has made great advances, gradually entering the era of personalized medicine 5. Platinum-based therapy addition with docetaxel, bevacizumab, ramucirumab or necitumumab produced a moderate improvement in the survival of NSCLC patients 6-9. Although these therapies are intended to be curative, they have great limitations, such as drug resistance, side effects and high recurrence rate 2. Therefore, continued research into new targets for molecular diagnosis and targeted therapy of NSCLC is required.

Tumor microenvironment is one of the important factors affecting tumor behavior, and characterized by dynamic changes as the tumor progresses 10. Accumulating evidence have shown that immune cells infiltrated in the tumor microenvironment can become therapeutic targets 11-14. In particular, tumor-infiltrating lymphocytes are associated with chemotherapy response and good prognosis 15, 16. Importantly, given that the programmed death 1(PD-1)/programmed death ligand 1 (PD-L1) induces immune escape between tumors and T lymphocytes, the emergence of PD-1 inhibitors provides new possibilities for targeted cancer therapy 17-19. Therefore, the identification of novel molecule targets involved in tumor behavior is essential for the treatment of cancer.

CD52 is a glycoprotein that consists of 12 amino acids and an amino acid terminal oligosaccharide linked to asparagine and is anchored to the cell membrane by glycosyl phosphatidylinositol (GPI) 20. In addition, CD52 is widely expressed in lymphocytes, monocytes, and dendritic cells, especially CD4+, CD16+, T cells, B cells, and bone marrow cells 21. Furthermore, CD52 was initially identified as a target of CAMPATH rat monoclonal antibody for lymphocyte-scavenging 22, 23. The monoclonal antibody Alemtuzumab combined with CD52 was used to treat chronic lymphocytic leukemia, multiple sclerosis and other autoimmune diseases 24. Recently, it has been reported that CD52 is a prognostic biomarker related to the tumor microenvironment of breast cancer 25. Nevertheless, the role of CD52 in solid tumors has not been clearly revealed.

In this study, immunohistochemical (IHC) staining analysis was performed to determine the expression pattern of CD52 in NSCLC. Moreover, loss of function assays were used to evaluate the biological functions of CD52 in NSCLC cells *in vitro* and *in vivo*. Collectively, the clinical significance and biological function of CD52 in the malignant behavior of NSCLC were clarified, which provides a reliable theoretical basis for precise treatment of NSCLC.

## Materials and Methods

### Ethical statement and clinical sample collection

This study was approved by the Ethics Committee of The Second Affiliated Hospital of Nanchang University. All included cases were pathologically diagnosed as NSCLC without any preoperative radiotherapy or chemotherapy, which included tumor tissues (n=79) and matched normal tissues (n=88). Inclusion criteria were patients between 18 and 80 years of age with histologically or cytologically confirmed NSCLC who had signed informed consent. The exclusion criteria were: 1) Patients had other types of cancer. 2) The patient has a serious disease, such as cardiovascular disease, liver disease, kidney disease, autoimmune disease or other. 3) Patients had received radiotherapy, chemotherapy, or immunotherapy for NSCLC prior to enrollment. 4) The patient is pregnant or lactating. 5) Patients with serious mental disorders, unable to cooperate with the signing of informed consent.

### Immunohistochemical (IHC) staining analysis

The tissue samples were dewaxed with xylene for 15 min each time, hydrated with 100% ethanol for 10 min, and added with PBS-H_2_O_2_ containing 0.1% Tween 20. After the samples were washed three times with PBS, they were incubated with primary antibody (anti-CD52, 1:100, Abcam, ab234412) overnight at 4 °C and continue the incubation with secondary antibody IgG (1: 400, Abcam, ab6721) overnight for 2 h at room temperature in a humidified chamber. Afterwards, the tissue samples were stained with DAB (3, 3'-diaminobenzidine) and hematoxylin. The staining scores of CD52 were evaluated by two independent pathologists based on both the proportion of positively stained tumor cells and the intensity of staining. The proportion of positive tumor cells was scored as follows: 0: no positive tumor cells; 1: <10%; 2: 10%-35%; 3: 35%-75%; 4: >75%. Staining intensity was graded as follows: 1, no staining; 2, weak staining (light yellow); 3, moderate staining (yellow brown); 4, strong staining (brown). In addition, a score higher than the median was defined as high expression of CD52, or low expression of CD52 for scores lower than the median.

### Cell culture

NSCLC cell lines (A549 and NCI-H1299) and normal pulmonary bronchial epithelial BEAS-2B cells were purchased from the Shanghai Cell Bank (Shanghai, China). All the cell lines were cultured in RPMI-1640 supplemented with 10% fetal bovine serum (FBS), 100 IU/mL penicillin, and 100 μg/mL streptomycin, which were maintained in a humidified environment of 5% CO_2_ at 37 °C. Notably, these cells were tested for mycoplasma contamination.

### Cell transfection

The short hairpin RNA (shRNA) oligos targeting CD52 (shCD52) and a non-targeting scrambled sequence (shCtrl, as negative control) were synthesized, as follows: shCD52-1: 5'-GGCCAATGCCATAATCCACCT-3', shCD52-2: 5'-CAGATACAAACTGGACTCTCA-3', shCD52-3: 5'-CCTCAGCATCCAGCAACATAA-3'; shCtrl: 5'-TTCTCCGAACGTGTCACGT-3'. The synthesized fragment was attached to the lentiviral vector BR-V108 labeled with green fluorescent protein (GFP) (Bioscienceres). Next, 3×10^8^ TU/ml lentivirus particles containing the target gene sequence (shCD52 or shCtrl) was transfected into A549 and NCI-H1299 cells using Lipofectamine^®^ 3000 (Thermo Fisher Scientific) with 20 multiplicity of infection (MOI) for 40 min at 37°C. After cultured for 72 h, GFP expression was observed under a fluorescence microscope (Olympus) and the stable cell lines were selected with puromycin as previously described 26.

### Quantitative real time PCR (qRT-PCR)

The cells' (A549, NCI-H1299 and BEAS-2B) RNA was extracted with Trizol (Invitrogen) and the concentration and quality were determined by a Nanodrop 2000/2000 C spectrophotometer (Thermo Fisher Scientific). The RNA was synthesized to the corresponding cDNA using Promega M-MLV Kit (Vazyme) according to the manufacturer's protocols. The qRT-PCR was performed in a two-step method and melting curve was produced by using AceQ qPCR SYBR Green Master Mix (Vazyme). The primer sequence as follows, CD52: 5'-CGCTTCCTCTTCCTCCTAC-3' and 5'-TTTGGCTGGTGTCGTTTT-3'; GAPDH (reference control): 5'-TGACTTCAACAGCGACACCCA-3' and 5'-CACCCTGTTGCTGTAGCCAAA-3'.

### Western blotting (WB) analysis

To determine the protein expression of CD52 in A549 and NCI-H1299 cell lines, WB analysis was performed as previously described. In brief, cell protein was extracted with lysis buffer (Sigma-Aldrich), supplemented with protease inhibitor (Thermo Fisher Scientific) and phosphatase inhibitor (Roche) and protein concentrations were quantified using BCA Protein Assay Kit (Pierce). Equal amounts of protein samples were resolved on 10% SDS-PAGE and transferred to polyvinylidene fluoride (PVDF) membranes (Millipore). After the membranes were blocked with 5% BSA in TBST for 1 h at room temperature, they were incubated with primary antibody (anti-CD52, 1:1000, Abcam, ab234412; anti-AKT, 1:3000, CST, 4691S; anti-p-AKT, 1:2000, Proteintech, 66444-1-Ig; anti-mTOR, 1:3000, Proteintech, 66888-1-Ig; anti-p-mTOR, 1:2000, Proteintech, 67778-1-Ig; anti-Bcl-2, 1:2000, Abcam, ab182858; anti-Bcl-w, 1:1000, CST, 2724; anti-CCND1, 1:2000, Abcam, ab134175; anti-c-Myc, 1:1000, CST, 13987S; GAPDH, 1:3000; Bioworld, AP0063) overnight at 4°C and continue the incubation with secondary antibody IgG (1:3000, Beyotime, A0208 or A0216) overnight for 2 h at room temperature in a humidified chamber. Immunoreactive proteins were visualized using the enhanced chemiluminescence ECL+Plus^TM^ detection Kit (Amersham Pharmacia Biotech) according to the manufacturer's instructions.

### Cell counting assay

A549 and NCI-H1299 cells were seeded into 96-well plates with a cell density of 2,000 cells per well and further cultured in an incubator with 5% CO_2_ at 37°C for 24 h. The cell numbers were quantified once a day for 5 days using Celigo image cytometer (Nexcelom Bioscience, Lawrence, MA, USA) and created cell growth curves for each group.

### Colony formation assay

A549 and NCI-H1299 cells were inoculated on 6-well plates at a density of 1,000 cells/well for continue culture. When the number of cells was greater than 50 or the cell size was between 0.3 ~ 1.0 mm, it was considered as a single clone. At room temperature, the cells were fixed with 1 ml 4% paraformaldehyde for 50 min and stained with 500 μL Giemsa for 20 min. After washing the cells several times with double distilled water, photographed with the camera (SONY) and counted the number of cells.

### Apoptosis detection based on flow cytometry

A549 and NCI-H1299 cells were inoculated on 6-well plates at a seeding density of 1,000 cells/well for a week, washed with PBS, centrifuged at 12000 g for 1 min at 4°C and resuspended. Subsequently, 500 μL of diluted 1X Annexin V binding buffer (Thermo Fisher Scientific) was added and the cells were stained with 10 μL Annexin V-APC for 15 min at room temperature in the dark. Next, 1X binding buffer was added and assessed cell apoptosis ratio using the FACSCanto II flow cytometer (Guava easyCyte HT, Millipore).

### Cell migration assay

A549 and NCI-H1299 cells were inoculated on 6-well plates at a seeding density of 5×10^4^ cells/well and replaced serum-free medium. For wound-healing assay, the cell layer was scratched using the scratch instrument (cVP408FH; V&P Scientific) and scanned the 96-well plate using Cellomics (Thermo) under fluorescence-based Cellomics ArrayScan VTI analyzer (Thermo Fisher Scientific) (50×magnification) at 0 h and 24 h. The cell migration rate of each group was calculated according to the following formula: The migration ratio =[(S3+S4) - (S1+S2)] × 100%. Where S1+S2 is the area of the wound measured immediately at 0 h, and S3+S4 is the area of the wound measured at 24 h after the scratch were performed.

For Transwell assay, the inner chamber contained 100 μL of serum-free medium and the external chamber contained 600 μL 30% fetal bovine serum. The cells were added to each chamber for 24 h cultivation, the migrating cells were fixed with 4% formaldehyde and stained using Giemsa. Finally, the cells were randomly selected from five fields and photographed under a 200× microscope (Olympus).

### Aerobic glycolysis analysis

Glucose consumption, which was detected using the Glucose Uptake Cell-Based Assay Kit (Cayman, USA), and lactate production, which was measured using the Lactate Assay Kit-WST (Dojindo, Shanghai, China), were also quantified to detect the glycolysis level in A549 and NCI-H1299 cells. ATP synthesis was measured by fluorometric detection using the ATP Assay Kit (Ex/Em = 535/587 nm) (Abcam), according to the manufacturer's instruction. In addition, oxygen consumption rates (OCR) and extracellular acidification rates (ECAR) were considered as parameters to evaluate glycolysis stress and cell mitochondrial stress in pancreatic cancer cells using the Seahorse XF96 Glycolysis Analyzer (Seahorse Bioscience, MA, USA).

### Animal xenograft model

Animal experiments was conducted in accordance with guidelines and protocols for animal care and protection. Twenty 4-week-old male BALB/c nude mice (SLAC Laboratory, Shanghai, China) were housed at pathogen-free condition and randomly divided into two groups (shCtrl and shCD52, n=5 for each group) before experiments. A549 cells (200 μL, 1×10^7^ cells/ml) were injected subcutaneously into the right forelimb axillary in nude mice and monitored the tumor formation in real time. During this period, data on mouse body weight and tumor volume (π/6×L×W×W, where L represents the long diameter and W represents the short diameter) were recorded at 5-day intervals (days 8, 13, 18, 23, 30). On the last day of breeding (30th day), the mice were anesthetized via intraperitoneal injection of 0.7% pentobarbital sodium (10 μL/g) and tumor burden was assessed under the multi-spectral living imaging system (Lumina LT, Perkin Elmer, Massachusetts, USA). Finally, the mice were sacrificed by cervical vertebrae and the tumor tissues were subjected to hematoxylin and eosin (H&E) and IHC staining for detection of Ki67 expression (anti- Ki67, 1:200, Abcam, ab16667).

### Human Phospho-kinase Array assay

After CD52 knockdown in NCI-H1299 cells, the phosphorylation level of related proteins in the phosphorylated kinase signaling pathway was initially evaluated using human phosphor-kinase array kit (Bio-Techne, Shanghai, China) according to its manufacturer's instructions. Briefly, the cell lysates of NCI-H1299 cells were prepared and transferred into membrane, followed blocked with buffer for 24 h at 4°C. After being washed with PBST, the membrane was incubated with diluted detection antibody cocktail overnight at 4°C. Next, the washed membranes were incubated with streptavidin-HRP for 30 min at 4°C. Finally, the pixel density on the membrane was detected by Image Lab 6.0 software (Bio-Rad, USA).

### Statistical analysis

All data were analyzed using GraphPad Prism 8.0 software (GraphPad Software Inc., San Diego, USA) and P values < 0.05 were considered statistically significant. Statistical differences between two groups were analyzed by paired t-test or one-way ANOVA followed by Bonferroni's post hoc test analysis. Correlations between CD52 expression and clinicopathological factors of patients were analyzed using the Mann-Whitney U. Data represent at least three independent experiments.

## Results

### CD52 expression is elevated in NSCLC and correlates with poor prognosis

**Figure 1:**
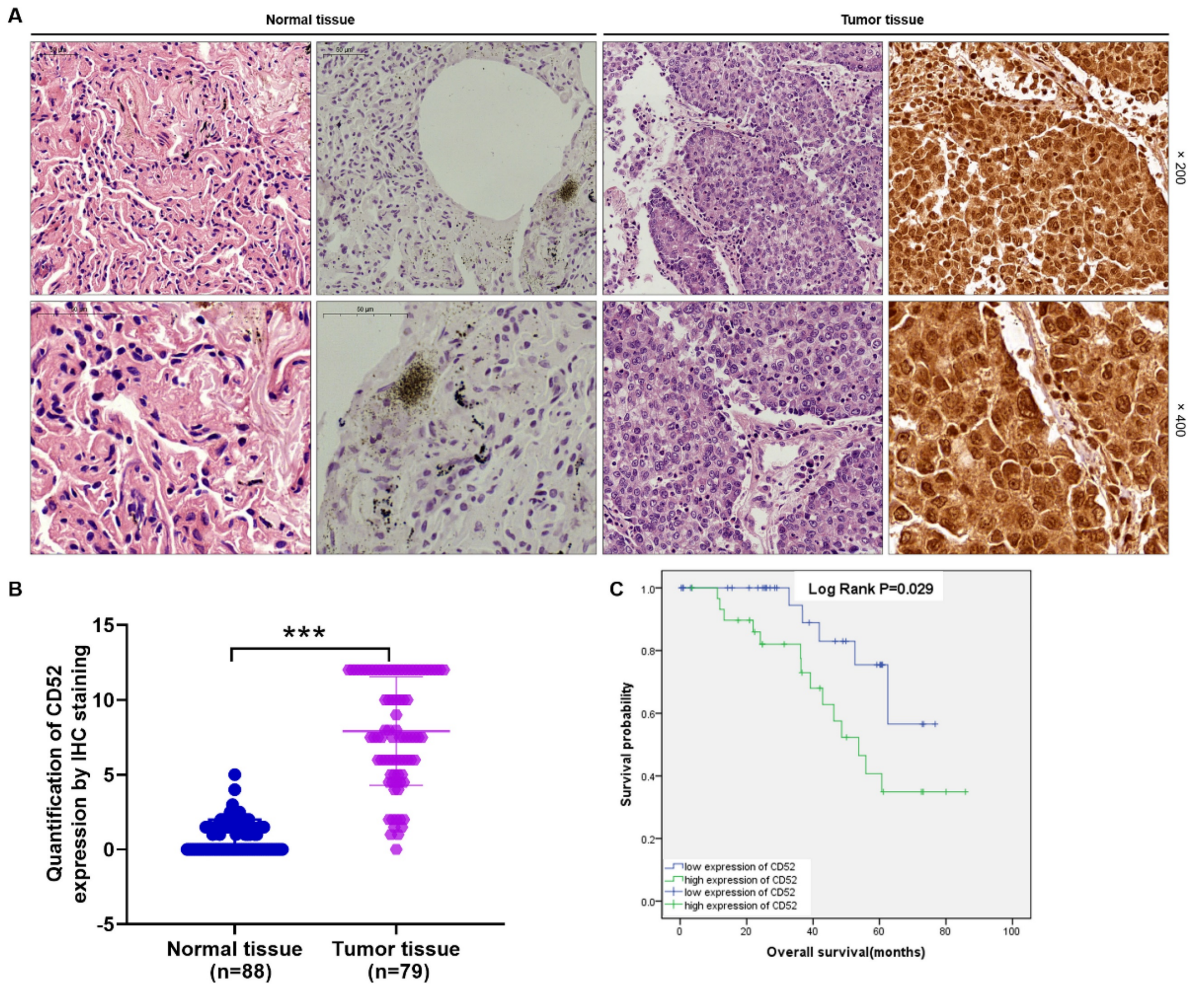
** CD52 was highly expressed in NSCLC.** (A) HE staining and IHC staining were used to analyze the expression of CD52 in tumor tissues and corresponding normal tissues of NSCLC patients. (B) According to the clinical information of NSCLC samples, the effect of CD52 expression level on survival time was analyzed by Kaplan-Meier method.

The CD52 expression level was determined using IHC staining in tumor tissues (n=79) and matched normal tissues (n=88) samples from clinical NSCLC patients. Hematoxylin and eosin (HE) staining showed the tissue structure and the number of nuclei (Fig. 1A). According to the scores of IHC staining, those with a median of 3 were considered as high CD52 expression, otherwise it was low CD52 expression. Excluding the tissues that failed in the IHC staining analysis, the high expression of CD52 accounted for 46.8% (37/79) of tumor tissues, while 0% (0/88) of normal tissues (p < 0.001) (**Supplementary Material-Table S1**). Consistently, the typical representative images of IHC staining showed that the number of CD52 positive cells in tumor tissues is significantly higher than that in normal tissues. In addition, the quantitative analysis results of IHC staining confirmed the above results (Fig. 1A). Moreover, Mann-Whitney U analysis based on clinicopathological data found that the expression level of CD52 was significantly correlated with stage (p = 0.003), and metastasis (M; p = 0.007) (Table 1). Similarly, Spearman correlation analysis again verified the above results, indicating that CD52 expression level was significantly positively correlated with NSCLC and stage, and metastasis (Table 2). Furthermore, high CD52 expression levels predicted a low probability of overall survival of patients with NSCLC (p = 0.014) (Fig. 1B). Collectively, CD52 expression was elevated in NSCLC and correlated with poor prognosis, which could be regarded as a potential diagnostic biomarker.

### Knockdown of CD52 inhibits the malignant phenotypes of NSCLC cells

CD52 expression was significantly higher in human NSCLC cell lines, including A549 and NCI-H1299, than in the normal pulmonary bronchial epithelia cell line BEAS-2B (p < 0.001) (Fig. S1A). To determine the function of CD52 in NSCLC, shRNA lentivirus system mediated knockdown of CD52 in A549 and NCI-H1299 cells were performed. Firstly, three shRNA oligos targeting CD52 (shCD52) sequences were synthesized and detected the knockdown efficiency of CD52 in NCI-H1299 cells. In view of the fact that the relative mRNA expression level of CD52 in shCD52-1 was the lowest compared to other groups (p < 0.01) (Fig. S1B), it was used for the subsequent construction of CD52 knockdown cell lines. Compared with the shCtrl group, the mRNA expression levels of CD52 in A549 and NCI-H1299 cells transfected with shCD52 were reduced by 81.84% (P < 0.05) and 70.9% (p < 0.01), respectively (Fig. S1C). Consistently, the CD52 protein expression level of these NSCLC cells was downregulated in shCD52 group (Fig. S1D).

**Figure 2:**
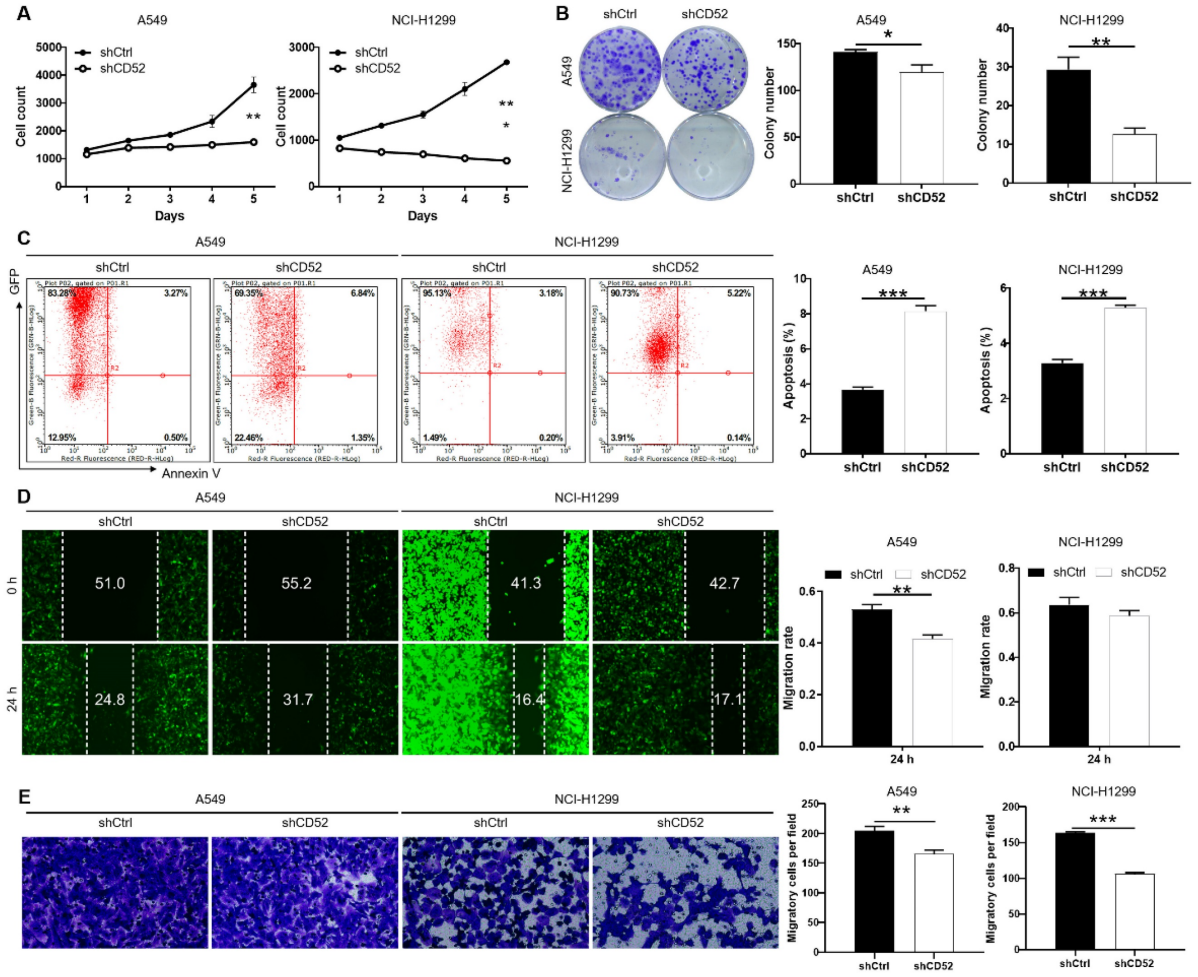
** Knockdown of CD52 inhibited proliferation and migration, enhanced apoptosis of NSCLC cells.** (A) Cell proliferation of A549 and NCI-H1299 cells with or without knockdown of CD52 was evaluated by cell counting experiment. (B) The effect of CD52 knockdown on the clone formation ability of A549 and NCI-H1299 cells was evaluated. (C) Flow Cytometry analysis based on Annexin V-APC staining was utilized to detect the percentage of apoptotic for A549 and NCI-H1299 cells. (D-E) Migration of A549 and NCI-H1299 cells with or without knockdown of CD52 was evaluated by wound-healing assay (D) and Transwell assay (E). The data were expressed as mean ± SD (n = 3), *P<0.05, **P<0.01, ***P<0.001.

The loss of function assays were used to evaluate the biological functions of CD52 in NSCLC cells *in vitro*. The results of cell counting assay indicated that CD52 knockdown by shRNA exhibited slower proliferation rate in A549 and NCI-H1299 cells than the negative group (p < 0.01) (Fig. 2A). Compared with the shCtrl group, the A549 and NCI-H1299 cells in the shCD52 group produced fewer and smaller cell clones (p < 0.05) (Fig. 2B). As expected, the results of flow cytometry demonstrate that apoptosis ratio of A549 and NCI-H1299 cells was significantly increased in shCD52 group than the shCtrl group (p < 0.001) (Fig. 2C). Furthermore, the effects of CD52 on the migration ability of NSCLC cells were evaluated by wound-healing and Transwell experiments. In the wound-healing assay, the migration rate of A549 cells in shCD52 group (24 h) was decreased by 22% than the shCtrl group (p < 0.01). However, the migration rate of NCI-H1299 cells was not significantly different between shCtrl and shCD52 groups (Fig. 2D). In addition, the results of Transwell experiments showed that the migration ability of NSCLC cells after CD52 knockdown was significantly inhibited (p < 0.01) (Fig. 2E). Moreover, CD52 knockdown downregulated protein expression of c-Myc, Bcl-2, Bcl-w, Cyclin D1 (CCND1) in NCI-H1299 cells (Fig. S2A).

Altogether, our data suggested that CD52 contributed to the cell proliferation and migration of NSCLC cells.

### Knockdown of CD52 inhibits the aerobic glycolysis of NSCLC cells

**Figure 3:**
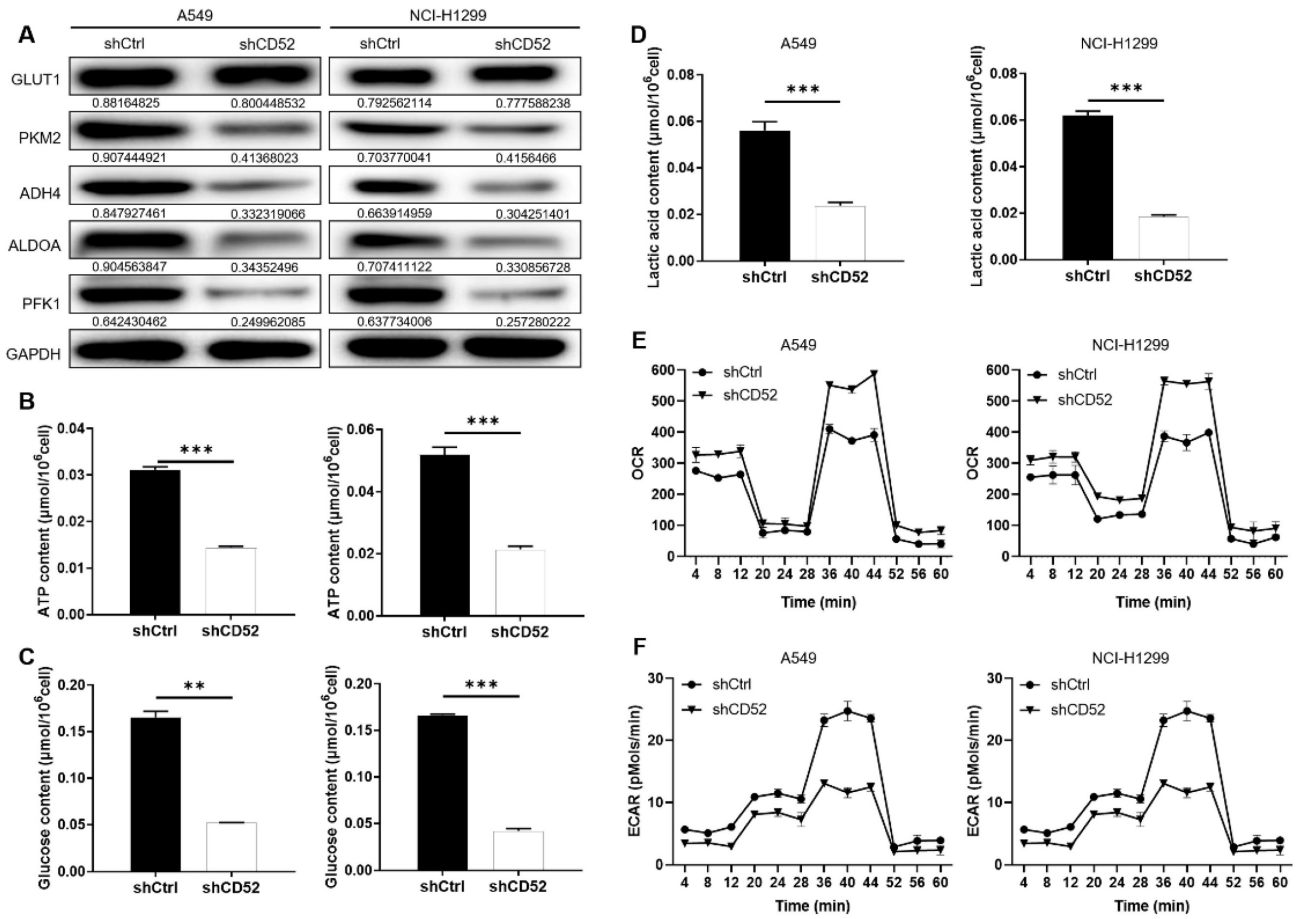
** Knockdown of CD52 inhibited glycolysis of NSCLC cells.** (A) The expression of glycolysis-related proteins in A549 and NCI-H1299 cells was detected by WB assay. (B) The effect of CD52 knockdown on the ATP content of A549 and NCI-H1299 cells was evaluated. (C) The effect of CD52 knockdown on glucose content in A549 and NCI-H1299 cells was observed. (D) The effect of CD52 knockdown on the lactic acid content of A549 and NCI-H1299 cells was observed. (E-F) The effects of CD52 knockdown on oxygen consumption rates (OCR) and extracellular acidification rates (ECAR) of A549 and NCI-H1299 cells were determined by kit method. The data were expressed as mean ± SD (n = 3), *P<0.05, **P<0.01, ***P<0.001.

Considering the importance of glycolysis in cancer, whether CD52 can regulate aerobic glycolysis in NSCLC cells has attracted our attention. Firstly, the expression of glycolysis-related proteins in A549 and NCI-H1299 cells was detected by WB assay. Our data indicated that knockdown of CD52 downregulated expression of glycolytic-related proteins, such as GLUT1, PKM2, ADH4, ALDOA and PFK1 (Fig. 3A). Furthermore, the content of ATP, glucose and lactic acid is an important index to reflect glycolytic activity. A549 and NCI-H1299 cells with CD52 knockdown showed significantly lower levels of ATP (p < 0.01), glucose (p < 0.1) and lactate (p < 0.01) compared with control cells (Fig. 3B-3D). Moreover, A549 and NCI-H1299 cells with CD52 knockdown showed increased OCR levels compared to control cells, while ECAR showed the opposite (Fig. 3E-3F). Therefore, these data suggested that CD52 knockdown inhibited aerobic glycolysis activity in NSCLC cells.

### CD52 regulates aerobic glycolysis of NSCLC cells through the AKT pathway

**Figure 4:**
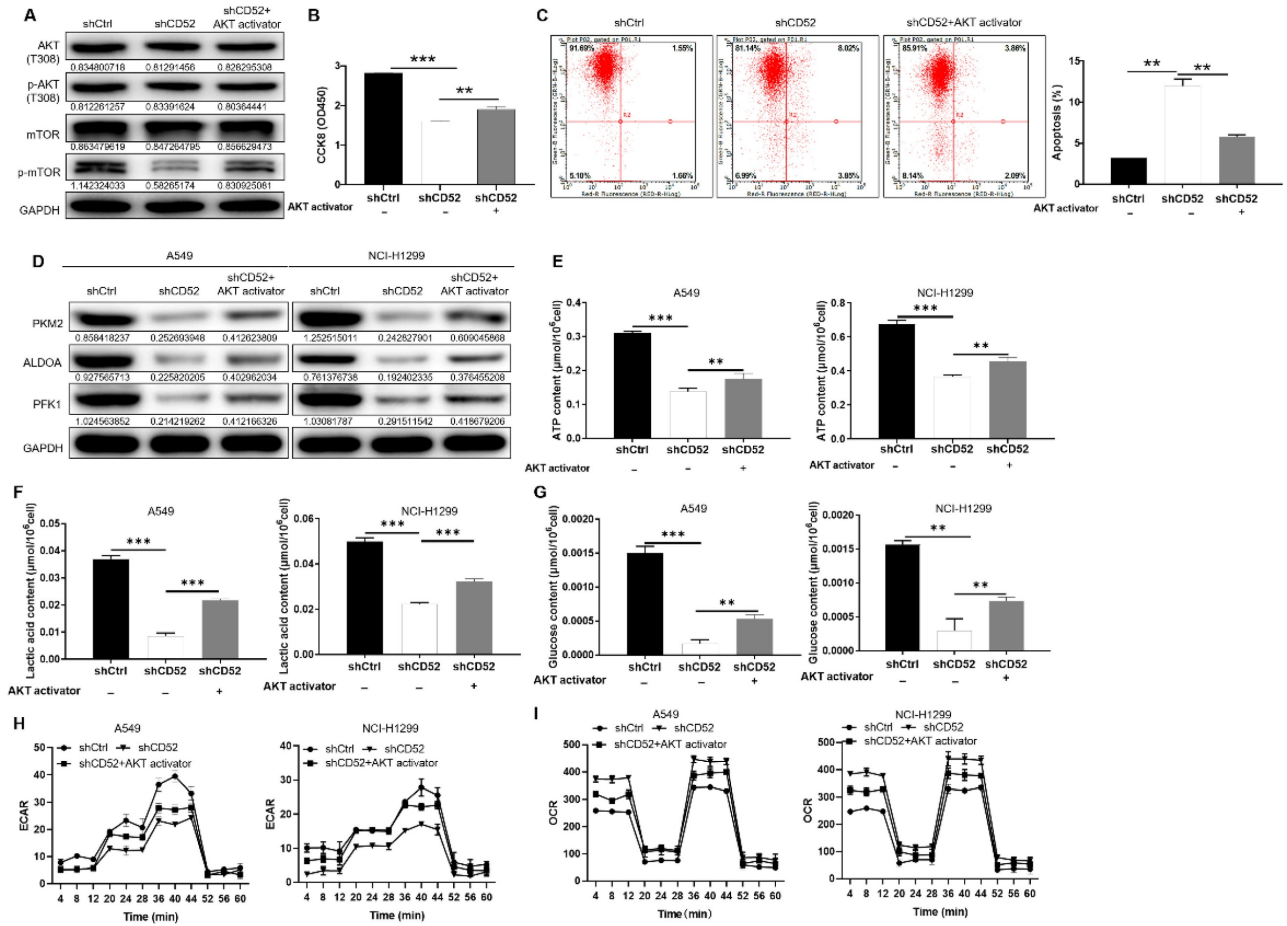
** CD52 regulates aerobic glycolysis of NSCLC cells through the AKT pathway.** (A-C) CD52 knockdown NCI-H1299 cells were treated with AKT activator to detect the alterations of AKT/p-AKT, mTOR/p-mTOR protein expression (A), proliferation (B) and apoptotic (C). (D) The expression of glycolytic related proteins in A549 and NCI-H1299 cells treated with AKT activator was detected by WB assay. (E) The effect of CD52 knockdown on the ATP content of A549 and NCI-H1299 cells was evaluated. (F) The effect of CD52 knockdown on the lactic acid content of A549 and NCI-H1299 cells was observed. (G) The effect of CD52 knockdown on glucose content in A549 and NCI-H1299 cells was observed. (H-I) The effects of CD52 knockdown on oxygen consumption rates (OCR) (H) and extracellular acidification rates (ECAR) (I) of A549 and NCI-H1299 cells were determined by kit method. The data were expressed as mean ± SD (n = 3), *P<0.05, **P<0.01, ***P<0.001.

After CD52 knockdown in NCI-H1299 cells, the phosphorylation level of related proteins in the phosphorylated kinase signaling pathway was initially evaluated. The knockdown of CD52 in NCI-H1299 cells upregulated the protein expression of AKT1/2/3 (S473), Chk-2 (T68), and STAT3 (Y705). On the contrary, the expression levels of AKT1/2/3 (T308), CREB (S133), STAT2 (Y689), and β-Catenin were significantly downregulated (p < 0.01) (Fig. S2B). WB results demonstrated that CD52 knockdown attenuated the phosphorylation levels of AKT (T308) and mTOR in NCI-H1299 cells, and this effect was partially reversed after AKT activator (20 μM) addition (Fig. 4A). Additionally, treatment with AKT activator partially alleviated CD52 knockdown effects on proliferation and apoptosis of NSCLC cells (p < 0.01) (Fig. 4B and 4C). Thus, CD52 regulated proliferation and apoptosis of NSCLC cells via AKT/mTOR signaling pathway.

Next, the CD52 knockdown A549 and NCI-H1299 cells were treated with AKT activator to detect glycolytic related markers, respectively. As shown in Fig. 4D, AKT activator treatment partially reversed the expression of PKM2, ALDOA, and PFK1. As previously mentioned, CD52 knockdown resulted in significant reductions in ATP, lactate, and glucose levels in NSCLC cells (p < 0.01), which were partially restored after AKT treatment (p < 0.01) (Fig. 4F-4G). After AKT treatment, ECAR decrease and OCR increase induced by CD52 knockdown can be partially restored (Fig. 4H-4I). Collectively, CD52 may regulate aerobic glycolysis of NSCLC cells through AKT pathway.

### Aerobic glycolysis induced by 2-DG inhibited the proliferation of NSCLC cells

**Figure 5:**
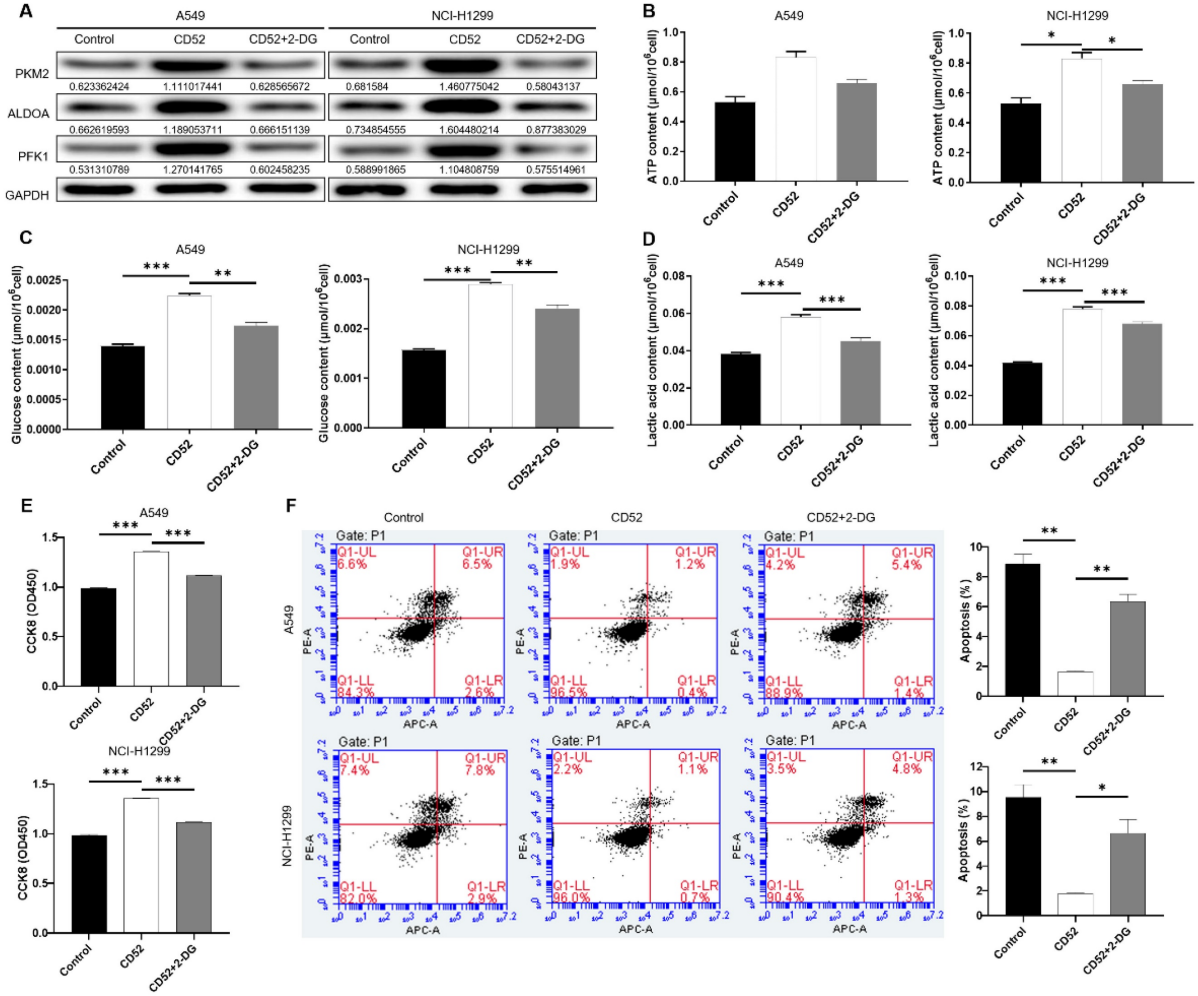
** CD52 affects the proliferation of NSCLC cells through aerobic glycolysis.** (A) The expression of aerobic glycolysis-related proteins in NSCLC cells overexpressed with CD52 was detected by WB assay after 2-DG treatment. (B-D) NSCLC cells with CD52 overexpression were treated with 2-DG and the contents of ATP (B), glucose (C) and lactic acid (D) were detected by the kit. (E-F) The proliferation (E) and apoptosis rates (F) of NSCLC cells overexpressed with CD52 were detected by 2-DG treatment. The data were expressed as mean ± SD (n = 3), *P<0.05, **P<0.01, ***P<0.001.

To determine whether CD52 regulates the malignant progression of NSCLC cells through aerobic glycolysis, we conducted the following experiments. CD52 overexpressed NSCLC cells were examined glycolytic related markers after being treated with 2-Deoxy-D-glucose (2-DG). As shown in Fig. 5A, overexpression of CD52 can upregulate the expression of PKM2, ALDOA and PFK1, while 2-DG treatment reduced the expression of these proteins (Fig. 5A). Additionally, CD52 overexpression resulted in significant increase in ATP, glucose and lactate levels in NSCLC cells (p < 0.01), which were partially restored after 2-DG treatment (Fig. 5B-5D). These data suggested that 2-DG treatment could reduce aerobic glycolysis in NSCLC cells. In addition, overexpression of CD52 enhanced proliferation and reduced apoptosis in both A549 and NCI-H1299 cells, while treatment with 2-DG reversed these effects accordingly. Therefore, our data indicated that aerobic glycolysis induced by 2-DG inhibited the proliferation of NSCLC cells.

### Knockdown of CD52 impairs tumor formation of NSCLC cells *in vivo*

**Figure 6:**
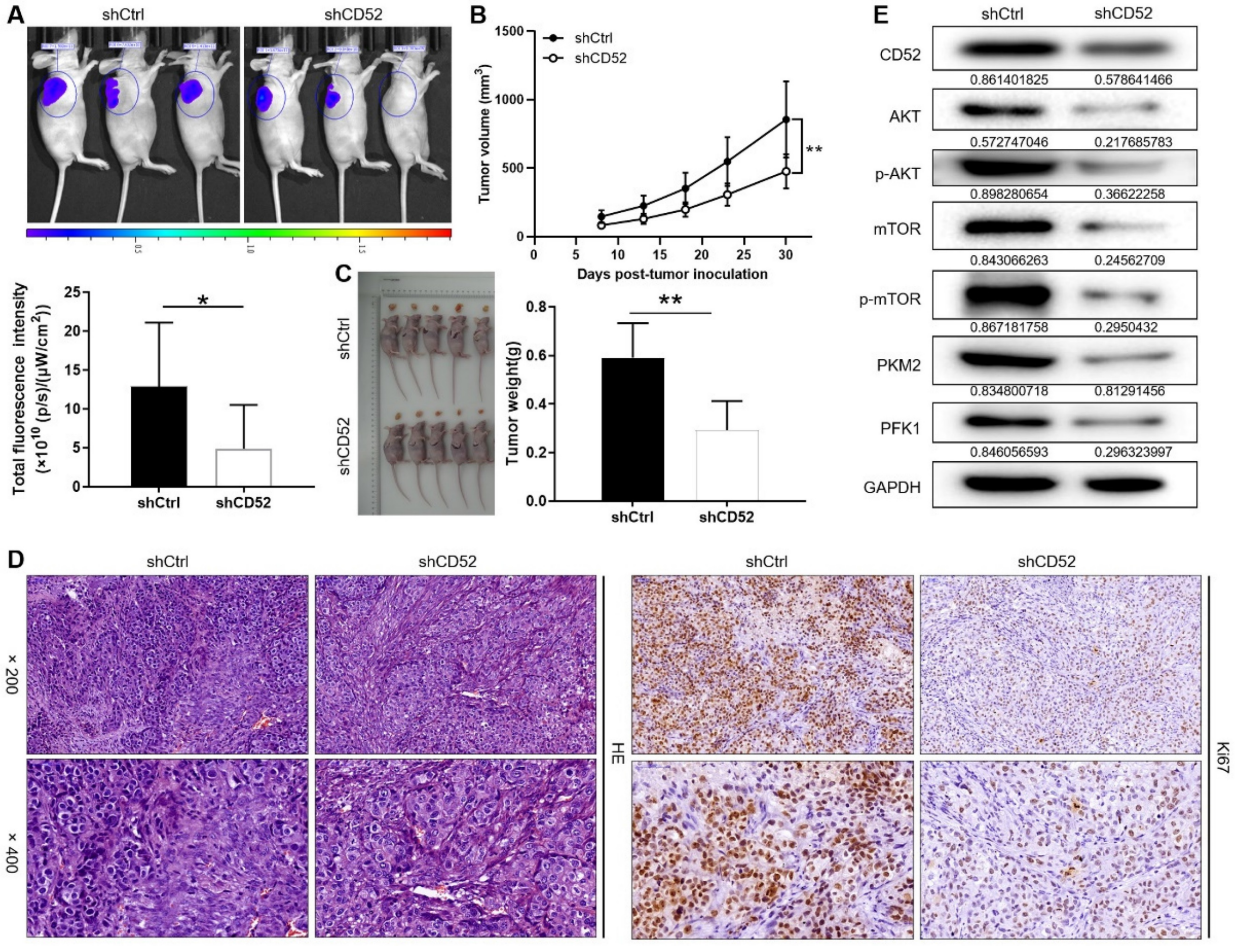
** Knockdown of CD52 suppressed tumor growth in mice xenograft models.** (A) The fluorescence intensity of mice tumors in shCtrl group and shCD52 group was detected, respectively. (B) The average volume of tumors in shCtrl group and shCD52 group was measured after post-injection. (C) The average weight of tumors in shCtrl group and shCD52 group was measured. (D) HE staining and IHC staining were used to analyze the expression of Ki67 between shCtrl and shCD52 groups. (E) The protein expression of shCtrl group and shCD52 group was detected by WB assay. The data were expressed as mean ± SD (n = 5), *P<0.05, **P<0.01.

A549 cells with the lentiviral vector that produced shRNA targeting CD52 (shCD52) and empty vector (shCtrl) were respectively inoculated into mice to construct nude mice xenograft models. On the last day of breeding (30th day), mice in the shCtrl and shCD52 groups were evaluated for the fluorescence intensity of the tumors. As illustrated in Fig. 6A, the tumor burden of mice after CD52 knockdown was significantly reduced (p < 0.05). The results of monitoring the growth of mice for 30 days showed in more detail that the tumor growth of the shCD52 group was significantly slower than that of the shCtrl group (Fig. 6B).

In detail, the tumors in the shCtrl group (the highest volume and weight were 1177.32 mm^3^ and 0.77 g) were significantly larger than those in the shCD52 group (the highest volume and weight were 612.40 mm^3^ and 0.42 g) (p < 0.01) (Fig. 6C). HE staining showed the tissue structure and the number of nuclei, confirming that the nodules in the mice were tumors. In addition, IHC staining showed that the Ki67 expression in xenograft tumors which developed from the CD52-knocked-down A549 cells was significantly downregulated as compared with that in tumors derived from shCtrl cells (Fig. 6D). Moreover, CD52 knockdown in mouse tumor tissues downregulated the expression of AKT pathway proteins and glycolytic-related proteins. Taken together, our results suggested that knockdown of CD52 possessed an inhibitory effect on tumorigenicity and glycolysis.

## Discussion

The emergence of targeted therapies and immunotherapy has dramatically changed the treatment of NSCLC, and longer survival is possible for some patients, even those with advanced disease 27, 28. Although some patients have a long-lasting response to these therapies, long-term patients may suffer significant toxicity and drug resistance 2. Moreover, gene expression characteristics, tumor genotypes, and tumor infiltrating lymphocytes in the tumor microenvironment seem to affect cancer progression and response to treatment 29, 30. In view of this, prediction and identification of new biomarkers for the treatment of NSCLC is essential.

More and more evidences confirmed that the tumor microenvironment innately modulated the occurrence, development and metastasis of tumors 10. In particular, immune cells infiltrating the tumor microenvironment participate in anti-tumor or tumor-promoting under the regulation of tumors 31. Therefore, the identification of key markers that regulate tumor immune microenvironment is essential for the regulation of tumor state. Interestingly, CD52, a glycophosphatidylinositol (GPI)-anchored glycoprotein, binds to the Siglec (sialic acid-binding Ig-like lectin)-10 receptor as a signaling molecule for immunosuppression on the surface of T cells, which inhibits the activation and proliferation of immune cells, especially CD4+ T cells 32. Furthermore, CD52 inhibits Toll-like receptor activation of NF-κB and triggers apoptosis to suppress inflammation 33. Clinically, CD52 is used as a highly immunosuppressive agent in chronic lymphocytic leukemia, lymphoma, multiple sclerosis and other autoimmune diseases 34, 35. In this context, the role of CD52 in NSCLC had aroused our great interest. In this study, we found that CD52 was significantly overexpressed in NSCLC compared to the corresponding normal tissues. In addition, CD52 expression was significantly correlated with tumor stage, T infiltration, tumor lymph node metastasis and distant tumor metastasis. These results indicated that CD52 may play an important role in the progress of NSCLC.

Dysregulation of cell proliferation and inhibition of cell death together provide a basic platform for tumor progression 36. Our findings suggested that downregulation of CD52 expression significantly suppressed the proliferation, migration and tumorigenesis of NSCLC cells. As we all know, c-Myc is a key regulator of cell proliferation, cell growth, differentiation, and apoptosis 37. Additionally, the antiapoptotic proteins of the Bcl-2 family, such as Bcl-2 and Bcl-w are key regulators of cell survival and are frequently overexpressed in malignancies, leading to increased cancer cell survival 38, 39. Furthermore, overexpression of Cyclin D1 (CCND1) leads to dysregulation of cyclin-dependent kinase (CDK) activity, rapid cell growth under conditions of restricted mitotic signaling and ultimately tumor growth 40. Consistently, our data indicated that CD52 knockdown downregulated protein expression of c-Myc, Bcl-2, Bcl-w and Cyclin D1 (CCND1) in NSCLC cells.

Moreover, the PI3K/AKT/mTOR signaling pathway is frequently activated and involved in proliferation, survival, invasion and metastasis in various human cancers 41. Many regulators of the PI3K/AKT/mTOR axis, including the catalysis of AKT, CREB, STAT2, mTOR and β-catenin have carcinogenic potential 41. This study revealed that the expression levels of AKT1/2/3 (T308), CREB (S133), STAT2 (Y689), and β-Catenin were significantly downregulated in NSCLC cells with CD52 knockdown. The results of WB indicated that CD52 knockdown decreased the phosphorylation level of AKT/mTOR in NSCLC cells. Furthermore, treatment with AKT activator partially reversed CD52 knockdown effects on proliferation and apoptosis of NSCLC cells. Taken together, CD52 regulates proliferation and apoptosis of NSCLC cells via AKT/mTOR signaling pathway. Nevertheless, the specific molecular mechanism of CD52 regulating NSCLC still requires further exploration.

## Conclusion

The expression of CD52 was significantly elevated in NSCLC and correlated with the patient prognosis. CD52 knockdown inhibited the malignant behaviors of NSCLC via the AKT/mTOR signaling pathway, which may be employed in an alternative therapeutic target for NSCLC.

## Supplementary Material

Supplementary figures and table.

## Figures and Tables

**Table 1:** Relationship between CD52 expression and tumor characteristics in patients with NSCLC.

Features	No. of patients	RPL35A expression		p value
		Low	High	
All patients	79	42	37	
Age (years)				0.437
<61 years	40	23	17	
≥61 years	39	19	20	
Gender				0.836
Male	46	24	22	
Female	33	18	15	
Tumor size				0.230
≤3cm	42	25	17	
>3cm	37	17	20	
Stage				0.003
I	25	16	9	
II	35	23	12	
III	13	3	10	
IV	6	0	6	
T Infiltrate				0.060
T1	42	25	17	
T2	19	11	8	
T3	6	5	1	
T4	12	1	11	
Lymphatic metastasis (N)				0.052
N0	35	21	14	
N1	36	21	15	
N2	8	0	8	
Metastasis				0.007
M0	73	42	31	
M1	6	0	6	

**Table 2:** Relationship between CD52 expression and tumor characteristics in patients with NSCLC.

		CD52
Metastasis	Spearman correlation coefficient	0.305
	Significance (double tail)	0.006
	N	79
Stage	Spearman correlation coefficient	0.335
	Significance (double tail)	0.003
	N	79
