# Supplementary material for: CD52 knockdown inhibits aerobic glycolysis and malignant behavior of NSCLC cells through AKT signaling pathway
Source: J Cancer. 2024 Apr 29;15(11):3394–405. doi: 10.7150/jca.86511 (PMC11134428; doi:10.7150/jca.86511)
Supplement: Supplementary file 1 — Supplementary figures and table. [file jcav15p3394s1.pdf]

Supplementary materials

Supplementary figures

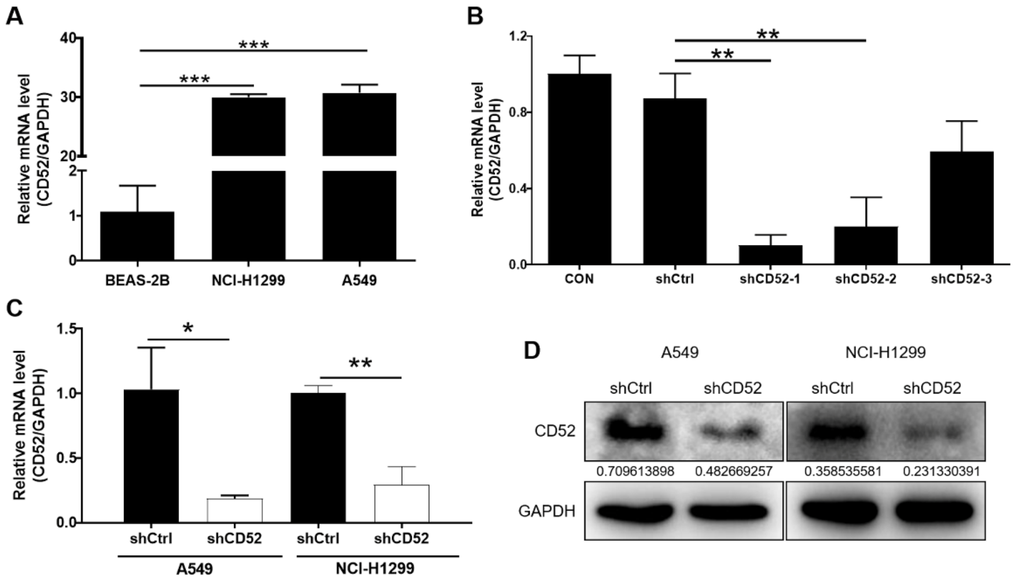

Figure S1. Construction of CD52 knockdown cell model. (A) The mRNA expression level of CD52 in NSCLC cell lines (A549 and NCI-H1299) and normal pulmonary bronchial epithelial BEAS-2B cells was determined. (B) The mRNA expression level of CD52 in short hairpin RNA (shRNA) oligos targeting CD52 (shCD52) sequence was detected. (C-D) The mRNA (C) and protein (D) expression levels of CD52 in A549 and NCI-H1299 cells transfected with lentivirus shCD52 and shCtrl were detected. The data were presented as the mean ± SD (n = 3), *P<0.05, **P<0.01, ***P<0.001.

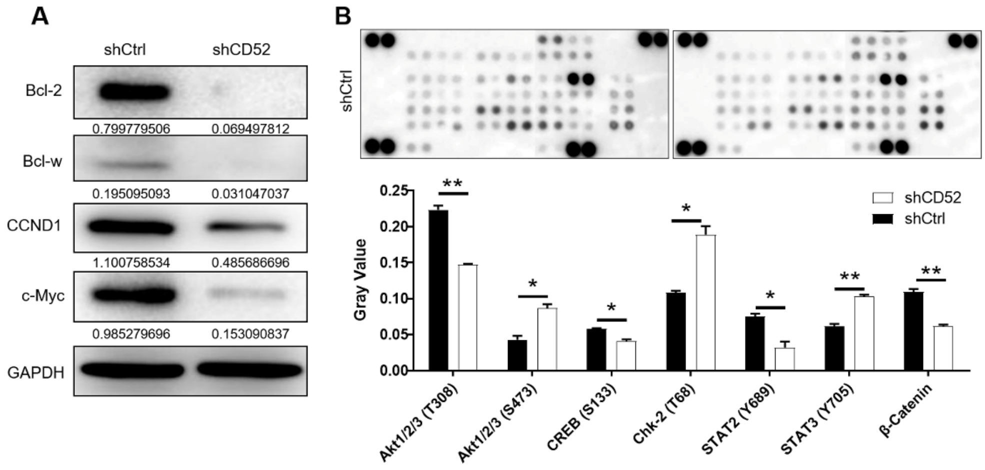

Figure S2. Exploration of downstream molecular mechanism of CD52 in NSCLC cells. (A) After CD52 knockdown in NCI-H1299 cells, the protein expression of Bcl-2, Bcl-w, CCND1 and c-Myc was analyzed using WB. (B) After CD52 knockdown in NCI-H1299 cells, the phosphorylation level of related proteins in the phosphorylated kinase signaling pathway was initially evaluated using human phosphor-kinase array kit. The data were expressed as mean ± SD (n = 3), *P<0.05, **P<0.01, ***P<0.001.

Supplementary tables

Table S1. Expression patterns in lung cancer tissues and para-carcinoma tissues revealed in immunohistochemistry analysis.

CD52 expression	Tumor tissue		Normal tissue		p value
	Cases	Percentage	Cases	Percentage	
Low	42	53.2%	88	100%	P<0.001
High	37	46.8%	0	0%	
